# Identification of a Tumor Microenvironment-Related Gene Signature Indicative of Disease Prognosis and Treatment Response in Colon Cancer

**DOI:** 10.1155/2021/6290261

**Published:** 2021-08-14

**Authors:** Wenzheng Chen, Jianfeng Huang, Jianbo Xiong, Pengcheng Fu, Changyu Chen, Yi Liu, Zhengrong Li, Zhigang Jie, Yi Cao

**Affiliations:** ^1^Department of Gastrointestinal Surgery, The First Affiliated Hospital of Nanchang University, China; ^2^Second Abdominal Surgery Department, Jiangxi Province Cancer Hospital, China; ^3^Medical College of Nanchang University, China

## Abstract

**Background:**

The tumor microenvironment (TME) is associated with disease outcomes and treatment response in colon cancer. Here, we constructed a TME-related gene signature that is prognosis of disease survival and may predict response to immunotherapy in colon cancer.

**Methods:**

We calculated immune and stromal scores for 385 colon cancer samples from The Cancer Genome Atlas (TCGA) database using the ESTIMATE algorithm. We identified nine TME-related prognostic genes using Cox regression analysis. We evaluated associations between protein expression, extent of immune cell infiltrate, and patient survival. We calculated risk scores and built a clinical predictive model for the TME-related gene signature. Receiver operating characteristic (ROC) curves were generated to assess the predictive power of the signature. We estimated the half-maximal inhibitory concentration (IC50) of chemotherapeutic drugs in patients using the pRRophetic algorithm. The expression of immune checkpoint genes was evaluated.

**Results:**

High immune and stromal scores are significantly associated with poor overall survival (*p* < 0.05). We identified 773 differential TME-related prognostic genes associated with survival; these genes were enriched in immune-related pathways. Nine key prognostic genes were identified and were used to construct a TME-related prognostic signature: CADM3, LEP, CD1B, PDE1B, CCL22, ABI3BP, IGLON5, SELE, and TGFB1. This signature identified a high-risk group with worse survival outcomes, based on Kaplan-Meier analysis. A nomogram composed of clinicopathological factors and risk score exhibited good accuracy. Drug sensitivity analysis identified no difference in sensitivity between the high-risk and low-risk groups. High-risk patients had higher expression of PD-1, PDL-1, and CTLA-4 and lower expression of LAG-3 and VSIR. Infiltration of dendritic cells was higher in the high-risk group.

**Conclusions:**

We identified a novel prognostic TME-related gene expression signature in colon cancer. Stratification of patients based on this gene signature could be used to improve outcomes and guide better therapy for colon cancer patients.

## 1. Introduction

Despite significant advances in our understanding of cancer biology and clinical cancer management, colon cancer remains a devastating disease. According to the Global Cancer Statistics 2018 [1], there were ~1.1 million new colon cancer cases and 551,269 deaths of colon cancer in 2018, and the mortality rate of colon cancer is expected to rise by 60% by the year 2035 [2]. In China, there were approximately 376.3 per 100,000 new cases and 191.0 per 100,000 cancer deaths of colon cancer in 2015, according to the data of the National Central Cancer Registry of China [3]. Several factors may help to explain the high mortality of colon cancer, including diagnosis at late stage, a high propensity for disease metastasis, and inherent drug resistance. Despite advances in options for colon cancer treatment, the 5-year survival rate remains at just 64.9% [4]. Therefore, it is urgent to discover additional diagnostic and prognostic biomarkers and therapeutic targets that can be used to improve long-term outcomes for patients with colon cancer.

The tumor microenvironment (TME), defined as the tissue environment surrounding a tumor, plays an important role in the tumorigenesis and prognosis of colon cancer. The TME contains not only cancer cells but also noncancer cells, including components of the extracellular matrix, blood vessels, immune cells, neurons, and cells that perform other tissue functions; these cell types all contribute to the malignant phenotypes of cancer, including uncontrolled proliferation, resistance to apoptosis, and evasion of immune surveillance [5, 6]. Thus, the composition and proportion of stromal and immune cells in the TME may significantly influence therapeutic responses and clinical outcomes in cancer patients [7, 8]. For example, the lung cancer lineage specifiers SOX2 and NKX2-1 contribute to tumor cell fate and neutrophil recruitment, suggesting that the determination of tumor immune microenvironment might impact the nature of the tumor [9]. Yamamoto et al. [10] performed a comprehensive multiplex immunohistochemistry analysis of the TME and found that M2 macrophage infiltration is useful to predict response to neoadjuvant chemotherapy and long-term survival in patients with esophageal cancer. Recently, several studies have proposed immune gene expression-based signatures for risk stratification and for predicting clinical outcomes in many kinds of cancers. For example, Cheng et al. [11] reported that an immune-related risk signature had prognostic significance in patients with glioblastoma and also showed that the immune status and local immune response could be indicated by the risk signature.

In the present study, we analyzed the association of immune and stromal scores with clinicopathological features of colon cancer. We identified a set of TME-related genes that could predict disease outcomes and treatment responses of patients with colon cancer. A combination of multiple immune biomarkers was developed to construct a novel prognostic and treatment-related signature which could be used to guide prognostic and therapeutic decisions for colon cancer patients. We also estimated the value of this signature to predict treatment efficacy in patients with colon cancer for a variety of treatments, including chemotherapy and immunotherapy. Overall, this study deepens our understanding of the impact of the tumor microenvironment on colon cancer outcomes and treatment responses and may be useful to provide a basis and reference for the clinical management and targeted therapy of colon cancer.

## 2. Materials and Methods

### 2.1. Raw Data Acquisition

Transcriptome RNA-seq data and corresponding clinical data for 385 colon cancer samples were downloaded from TCGA database. The clinical information of the patients is shown in Table 1.

### 2.2. Generation of Stromal and Immune Scores

The ESTIMATE algorithm [12] was used to evaluate the immune and stromal contents to provide immune and stromal scores for each colon cancer sample. Immune score and stromal score are positively related to the ratio of immune and stromal cells; the higher the respective score, the larger the ratio of the corresponding component in the TME. The samples were divided into the high and low score groups by the median of the scores.

### 2.3. Acquisition of Differentially Expressed Genes and Functional Enrichment Analysis

Differentially expressed genes (DEGs) between the high score and low score in the immune group and stromal groups were identified by using differential expression analysis in the limma package in R Statistical Software [13]. The DEGs were screened by the following threshold parameters: fold change > 1 and *p* < 0.05. Only terms with both *p* and *q* values < 0.05 were considered significantly different. DEGs were visualized by heatmaps with the pheatmap package in R. The intersection of differentially expressed immune-related and stromal-related genes was evaluated by a Venn diagram.

To explore the possible pathways resulted to the differences in the immune-related genes and the stromal-related genes, GO and KEGG enrichment analyses were performed using the packages clusterProfiler [14], enrichplot, and ggplot2 in R, with a statistical threshold set at *p* < 0.05.

### 2.4. Survival Analysis and Association of Scores with Clinical Stage

Survival analysis was performed using with the survival and survminer packages in R. Overall survival (OS) curves were plotted by the Kaplan-Meier method and differences evaluated using the log rank test. A *p* value < 0.05 was regarded as statistically significant. The clinicopathological characteristic data of colon cancer patients were downloaded from TCGA. Association of scores with clinicopathological features was carried out using R, and the Wilcoxon rank sum or Kruskal-Wallis rank sum test was performed as a test of significance.

### 2.5. Screening of Key Prognostic TME-Related Genes

To identify key prognostic genes associated with the TME, we performed univariate Cox regression analysis to estimate the relationship between each gene individually and the OS of patients. The top 17 DEGs were selected in a stepwise manner to perform multivariate Cox regression analysis. Ultimately, 9 DEGs were identified for a further study.

### 2.6. Immunohistochemistry (IHC) and Immune Cell Infiltration of the Nine DEGs

Images of immunohistochemistry (IHC) staining of the protein products of the 9 DEGs in colon cancer samples and para-cancer tissue were extracted from the Human Protein Atlas (HPA) database (http://www.proteinatlas.org).

The Tumor Immune Estimation Resource (TIMER) database [15] was utilized to explore the associations between the 9 DEGs and immune cell infiltration, including B cells, CD4+ T cells, CD8+ T cells, macrophages, neutrophils, and dendritic cells.

### 2.7. Construction of a TME-Related Gene Signature

We constructed a TME-related gene signature by using the generated coefficients and corresponding expression of the 9 selected DEGs. The formula for the gene signature risk score is as follows: risk score = *∑* (Exp (gene)∗coef (gene)), where Exp (gene) is the corresponding expression of the included genes and coef (gene) represents the regression coefficient from the multivariate Cox regression analysis. Patients were further classified into a low-risk group and a high-risk group according to the median risk score.

### 2.8. Validation of the TME-Related Gene Signature and Construction of a Nomogram

To verify the independent prognostic value of the immune-related gene risk signature on disease survival, univariate and multivariate Cox regression analyses were conducted. A nomogram including age, gender, stage, TNM classification, and risk score was used to calculate the total score. We assessed the 1-, 3-, and 5-year survival probabilities based on the nomogram. The 1-, 3-, and 5-year dependent ROCs were utilized to assess the performance of the nomogram.

### 2.9. Estimation of Immune Cell Fraction

The infiltration level and proportion of distinct immune cells in colon cancer samples from TCGA database were quantified using CIBERSORT and leucocyte signature matrix 22 (LM22) [16]. We evaluated differences in the immune cell fraction between the low-risk and high-risk groups.

### 2.10. Exploration of the Significance of the Model in Predicting Response to Chemotherapy, Targeted Therapy, and Immunotherapy

To evaluate the model in predicting the clinical response of colon cancer treatment, we calculated the IC50 of common chemotherapeutic and targeted agents, such as cisplatin, paclitaxel, sorafenib, and sunitinib. The differences in the IC50 between the high- and low-risk groups were compared by the Wilcoxon signed-rank test, and the results were shown as box drawings obtained using with pRRophetic and ggplot2 packages in R. To study the relationship between the model and the expression level of genes related to immune checkpoints, including PD-1, PDL-1, CTLA-4, LAG-3, and VSIR, we performed violin plot analysis using the ggstatsplot package in R.

### 2.11. Statistical Analysis

Spearman's correlation analysis was utilized to assess the correlations between the risk scores and immune cell infiltration and TME scores. Survival curves were conducted using the Kaplan-Meier method and the log-rank test. All statistical analyses were conducted using R Statistical Software version 4.0.4 and strawberry-perl-5.32.0.1. A *p* value < 0.05 was considered to indicate a significance difference.

## 3. Results

### 3.1. Immune and Stromal Scores, Screening of DEGs, and Functional Enrichment Analysis

A total of 385 cases with transcriptome RNA-seq data were downloaded from TCGA database to estimate the proportion of tumor-infiltrating immune cells and the amount of immune and stromal components in colon cancer samples. The clinical characteristics of the colon cancer cohort, including age, gender, clinical stage, and TNM classification, are presented in Table 1. We analyzed the selected patient data based on their gene expression profiles and calculated the immune scores and stromal scores by using the CIBERSORT and ESTIMATE algorithms. The immune scores ranged from -967.41 to 2405.66. The stromal scores ranged from -2204.16 to 1685.93.

**Figure 1:**
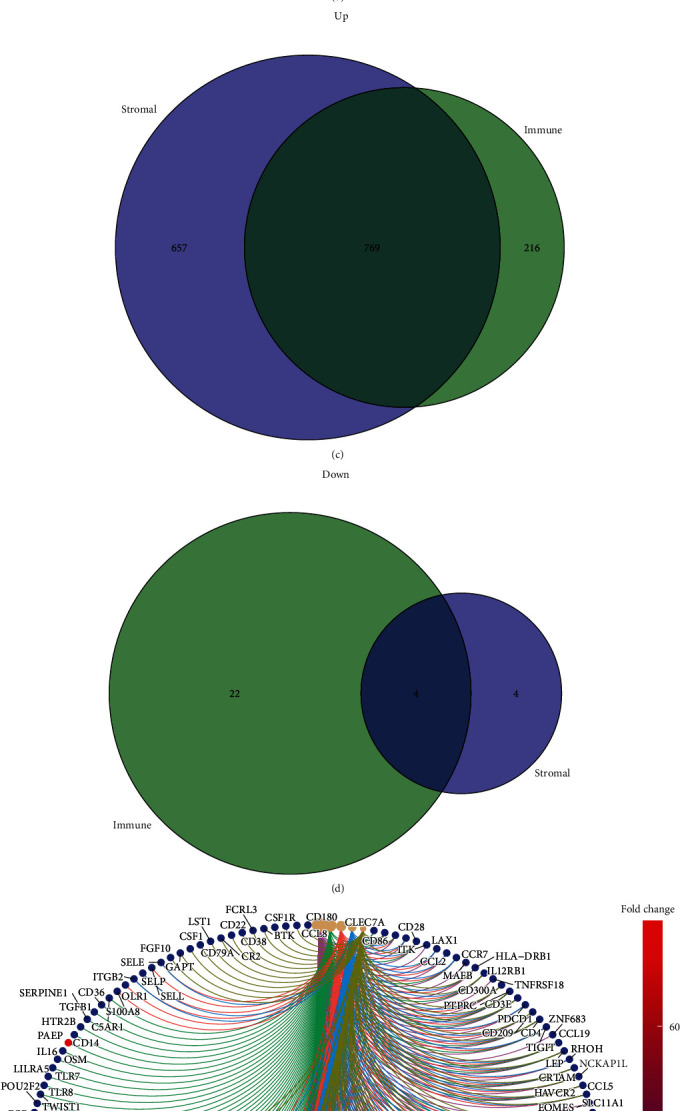
Differential gene expression analysis based on immune and stromal scores in colon cancer. (a, b) Heatmaps for DEGs identified by comparison of the high and low score groups for immune and stromal scores. (c, d) Venn diagrams for immune and stromal score overexpressed and underexpressed DEGs. (e, f) Circular plots for GO and KEGG enrichment analysis of the 773 DEGs.

A total of 1011 DEGs were identified between the samples with high and low immune scores; of these, 985 genes were overexpressed and 26 genes were underexpressed. Similarly, 1434 DEGs were obtained from samples with high vs. low stromal scores, including 1426 overexpressed and 8 underexpressed genes. We generated heatmaps to display the results of these comparisons between samples with high and low immune and stromal scores (Figures 1(a) and 1(b), respectively). Intersection analysis was performed to narrow the scope of target genes that are associated with immune and stromal score and identified 769 overexpressed and 4 underexpressed DEGs common to both comparisons (Figures 1(c) and 1(d)). These DEGs (total 773 genes) were selected for further analysis as potential factors that could determine the status of the TME.

Gene Ontology (GO) and Kyoto Encyclopedia of Genes and Genomes (KEGG) pathway enrichment analyses were performed to evaluate the possible functions of the 773 DEGs in the intersection. The results from GO enrichment analysis indicated that the DEGs mapped to the immune-related GO terms, including leukocyte cell-cell adhesion, leukocyte migration, positive regulation of cytokine production, regulation of lymphocyte activation, and T cell activation (Figure 1(e)). The results from KEGG enrichment analysis identified enrichment in genes associated with cytokine-cytokine receptor interaction, hematopoietic cell lineage, rheumatoid arthritis, *Staphylococcus aureu*s infection, and viral protein interaction with cytokine and cytokine receptor (Figure 1(f)). These results suggested that these genes may function in immune responses of colon cancer.

**Figure 2:**
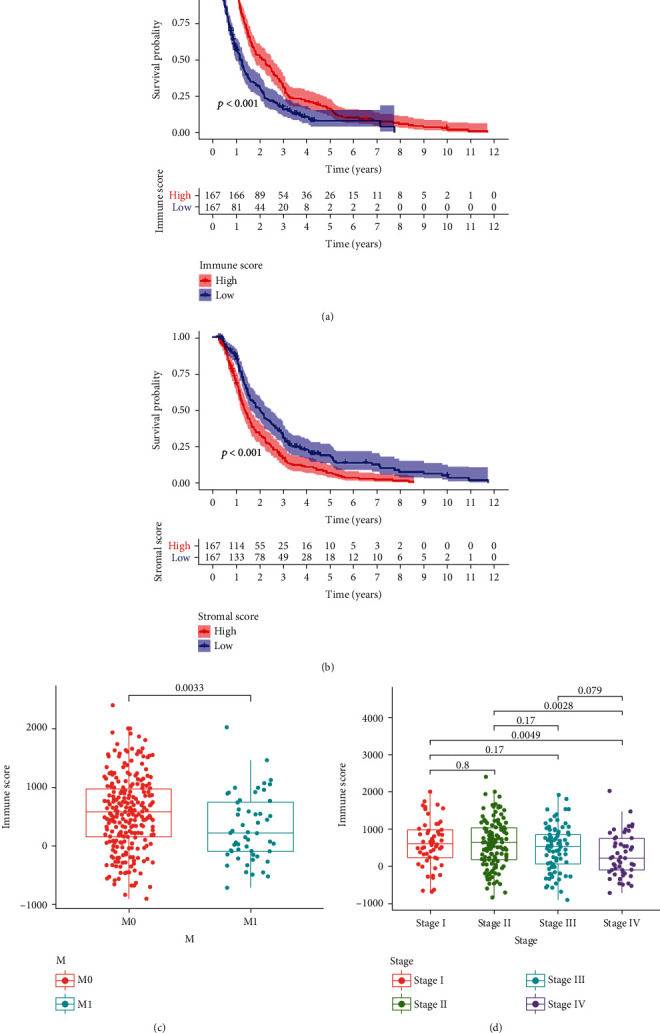
Association of immune and stromal scores with survival and M classification of colon cancer. (a) Kaplan-Meier survival analysis of colon cancer patients in the high and low immune score groups. (b) Kaplan-Meier survival analysis of colon cancer patients in the high and low stromal score groups. (c) Distribution of high and low immune scores in M classification. (d) Distribution of high and low immune scores in tumor stage.

Based on the median levels of the immune and stromal scores, the patient samples from TCGA cohort were, respectively, divided into the high and low groups. Survival curves based on the high and low immune and stromal scores were plotted to evaluate the prognostic value of the immune and stromal scores. Higher immune score was significantly associated with improved overall survival rate (Figure 2(a)). Higher stromal score was significantly associated with poorer overall survival rate (Figure 2(b)). High immune scores showed a significant association with advanced M classification of TNM classification (Figure 2(c)) and advanced clinical stages II and IV (Figure 2(d)). The association of stromal scores with TNM classification and clinical stage were not significant (Supplementary Figure 1). These results showed that the ratio of immune and stromal components is associated with disease prognosis in colon cancer.

### 3.2. Identification of Nine Immune-Related Key Prognostic Genes

**Figure 3:**
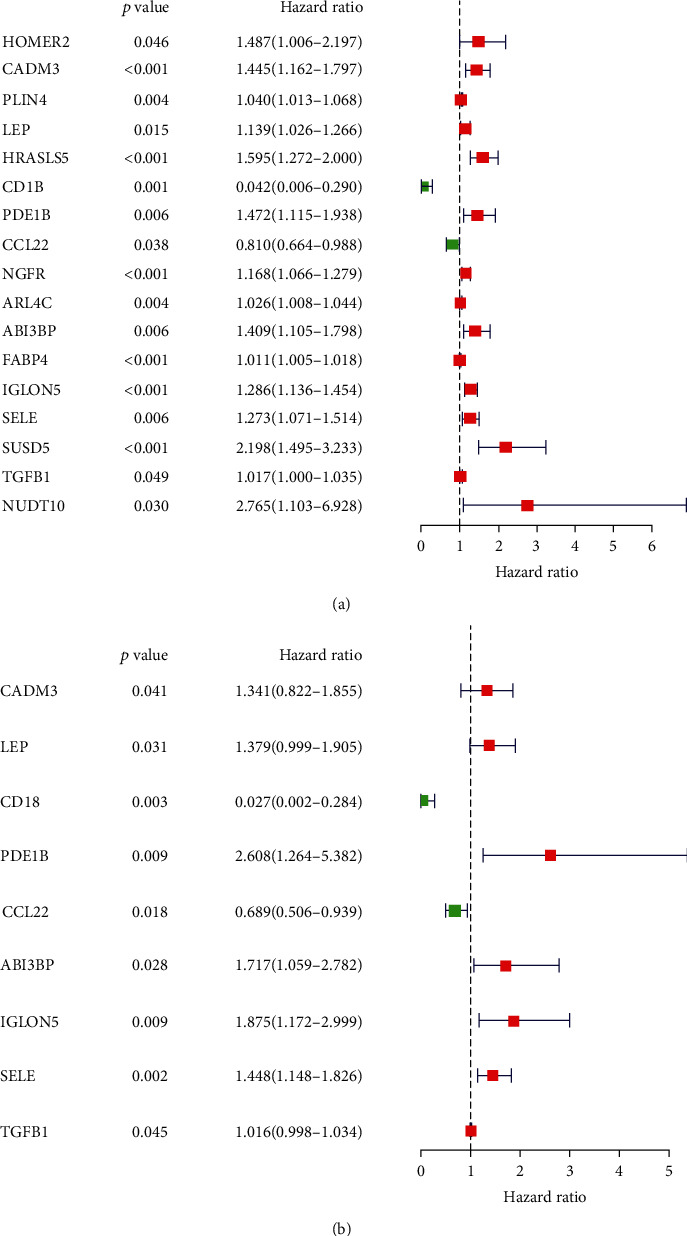
Identification of prognostic TME-related DEGs in colon cancer. (a) Univariate Cox analysis identified 17 TME-related DEGs that were significantly correlated with OS of colon cancer patients; forest plot showing the gene expression and prognosis of colon cancer patients. (b) Multivariate Cox analysis identified 9 TME-related DEGs that were significantly correlated with OS of colon cancer patients; forest plot showing the gene expression and prognosis of colon cancer patients.

Next, univariate Cox regression analysis and multivariate Cox regression were employed to explore the potential prognostic value of these DEGs in colon cancer outcomes. Univariate Cox regression analysis showed that 17 DEGs were significantly associated with survival (Figure 3(a)). Multivariate Cox regression analysis identified 9 DEGs (CADM3, LEP, CD1B, PDE1B, CCL22, ABI3BP, IGLON5, SELE, and TGFB1) that were significantly associated with survival (Figure 3(b)). The 9 genes were selected for further analysis. The coefficients of univariate Cox regression and multivariate Cox regression are presented in Tables 2 and 3.

### 3.3. Protein Expression and Prognostic Value of Nine Key Genes in Colon Cancer Patients

**Figure 4:**
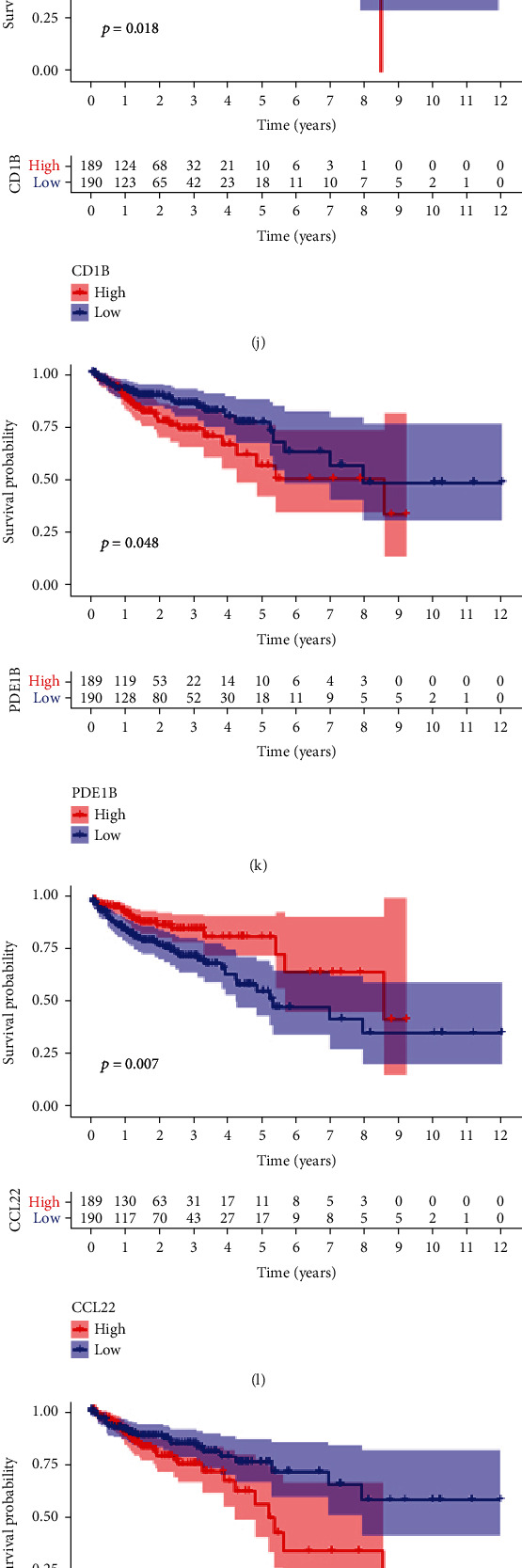
Immunohistochemical staining images from The Human Protein Atlas of seven key genes in colon cancer tissue (a–g). Differences in protein expression and OS analysis of the included DEGs in colon cancer (h–p).

We explored the protein expression of the 9 key genes in colon cancer using the Human Protein Atlas database. CCL22 and PDE1B were not available on the website, and we evaluated the expression of CADM3, LEP, CD1B, ABI3BP, IGLON5, SELE, and TGFB1 protein in this database. Staining for CADM3, IGLON5, and TGFB1 was low in tumor tissue; staining for LEP and ABI3BP was medium in tumor tissue; staining for CD1B was high in tumor tissue. SELE staining was negative in tumor tissue (Figures 4(a)–4(g)). Protein expression for all seven genes was found staining in tumor stromal tissue, suggesting that these seven genes may affect the tumorigenesis and prognosis of colon cancer by acting through stromal components.

The relationship of expression of these 9 key genes with prognosis of colon cancer patients was analyzed by the Kaplan-Meier method. Higher expression of CADM3, LEP, PDE1B, ABI3BP, IGLON5, SELE, and TGFB1 was significantly associated with shorter OS of colon cancer patients (Figures 4(h), 4(i), 4(k), and 4(m)–4(p)). High expression of CD1B and CCL22 was associated with longer OS for colon cancer patients (Figures 4(j) and 4(l)). These results indicated that the expression of the 9 key genes is significantly associated with the prognosis of colon cancer and suggested that these genes may be useful biomarkers for constructing a prognostic signature of colon cancer.

### 3.4. Association between Nine Key Genes and Immune Cell Infiltration Level in Colon Cancer

**Figure 5:**
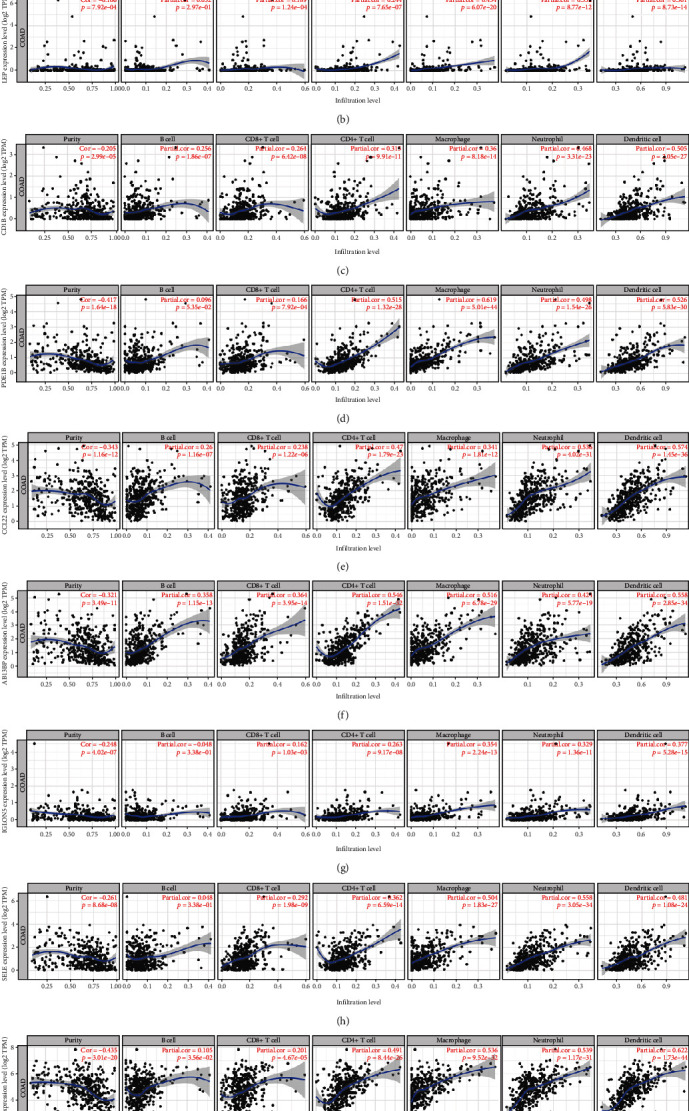
Correlation between the 9 DEGs and immune cell infiltration.

We explored the association between expression of the 9 key genes and immune cell infiltration in colon cancer using the TIMER database. Expression of CADM3, CD1B, PDE1B, CCL22, ABI3BP, IGLON5, SELE, and TGFB1 was related to immune cell infiltration (Figures 5(a) and 5(c)–5(i)). The expression of CD1B, CCL22, and ABI3BP was significantly positively correlated with levels of infiltrating B cells (Figures 5(c), 5(e), and 5(f)). Expression of CADM3, LEP, CD1B, CCL22, ABI3BP, SELE, and TGFB1 was significantly positively correlated with levels of infiltrating CD8+ T cells (Figures 5(a)–5(c), 5(e), 5(f), 5(h), and 5(i)). Expression of CADM3, LEP, CD1B, PDE1B, CCL22, ABI3BP, IGLON5, SELE, and TGFB1 was correlated to levels of infiltrating CD4+ T cells, macrophages, neutrophils, and dendritic cells.

### 3.5. Construction of a TME-Related Prognostic Signature

**Figure 6:**
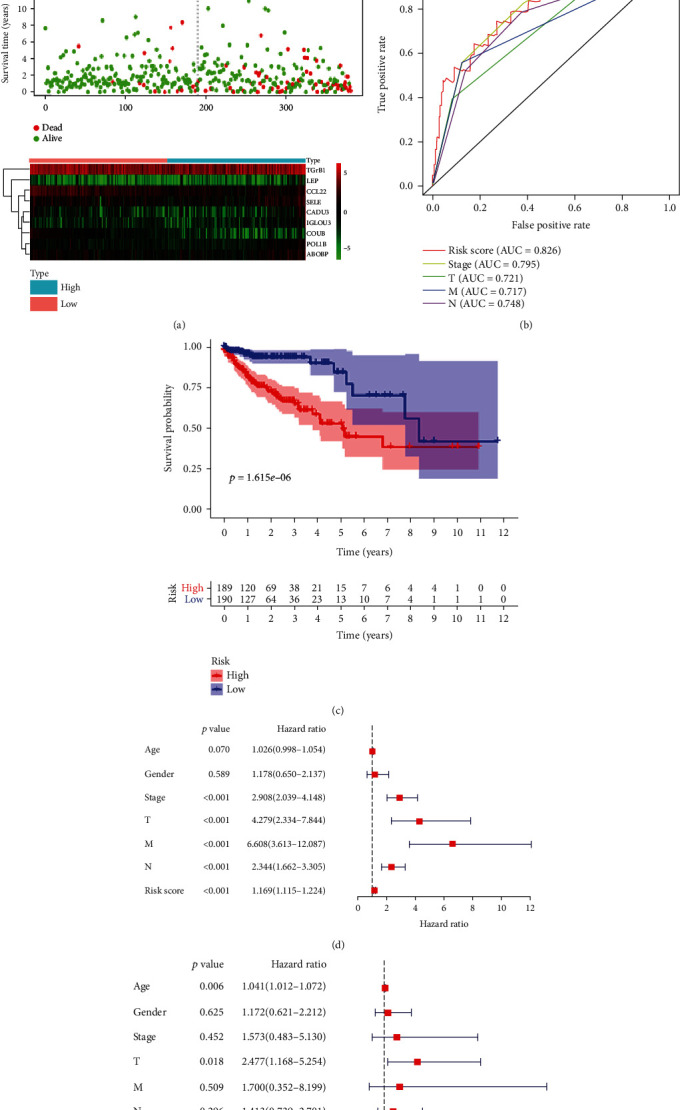
Construction of the TME-related gene signature and prognostic analysis. (a) Risk score distribution, survival status, and heatmap of gene expression among patients with colon cancer. (b) AUC values for risk score, stage, and T, N, and M classification. (c) Kaplan-Meier survival curve of OS in the high-risk and low-risk patient groups based on the signature. (d) Univariate and (e) multivariate Cox analyses of clinical characteristics and risk score with prognosis of patients with colon cancer in TCGA cohort.

A risk score was calculated based on the expression of all 9 key prognostic genes that were selected to establish a prognostic signature. For each patient sample, the risk score was calculated according to the followed equation: risk score = [(−1.076∗Exp (CADM3) + (0.321∗Exp (LEP)) + (−3.629∗Exp (CD1B)) + (0.958∗Exp (PDE1B)) + (−0.371∗Exp (CCL22)) + (0.5403∗Exp(ABI3BP)) + (0.628∗Exp(IGLON5)) + (0.370∗Exp(SELE) ) + (0.015∗Exp(TGFB1))] . The samples were divided into the high-risk and low-risk groups based on the median risk score. The distribution of risk score, the survival overview, and gene expression heatmap are presented in Figure 6(a). The area under the ROC curve for risk score, stage, and T, M, and N classification was 0.826, 0.795, 0.721, 0.717, and 0.748, respectively, which shows that the prognostic risk model demonstrates sensitivity and specificity comparable to or superior to other traditional prognostic factors (Figure 6(b)). Survival analysis showed that patients in the high-risk group had significantly poorer overall survival than those in the low-risk group (Figure 6(c)).

Univariate and multivariate Cox regression analyses were carried out stepwise to assess the predictive power of the prognostic signature. Univariate Cox regression analysis showed that stage; T, M, and N statuses; and risk core were significantly correlated with OS. The results of multivariate Cox regression analysis indicated that T status and risk score were independent prognostic factors for OS.

**Figure 7:**
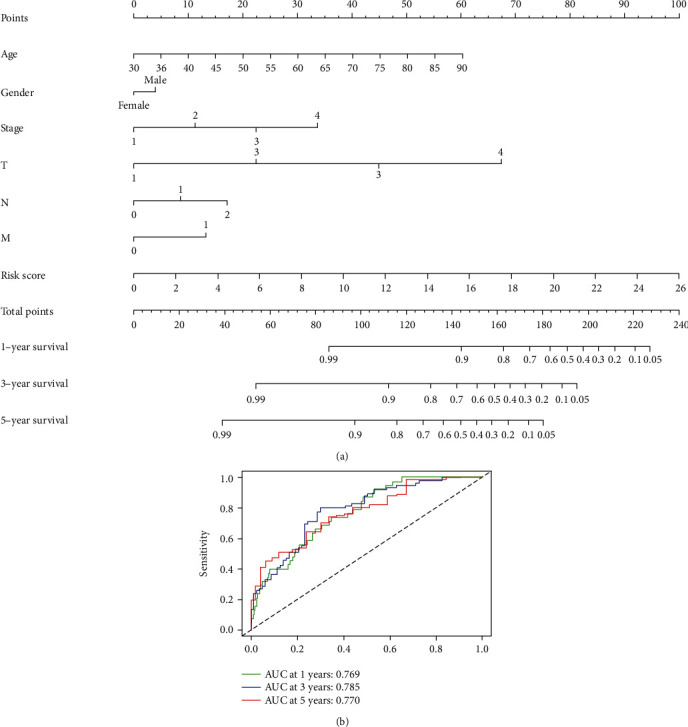
Construction of a nomogram and validation of the prognostic risk model in colon cancer patients. (a) A nomogram based on clinical characteristics and risk score among patients from TCGA cohort. (b) AUC values for 1-, 3-, and 5-year survival rates based on the nomogram.

A prognostic nomogram was established to provide a quantitative and visual method for predicting the 1-, 3-, and 5-year OS probability of colon cancer patients (Figure 7(a)). Based on this nomogram, the AUC values for 1-, 3-, and 5-year OS were 0.769, 0.785, and 0.770 (Figure 7(b)).

### 3.6. The Relationship between the Nine-Gene Signature and Infiltration of Immune Cells

**Figure 8:**
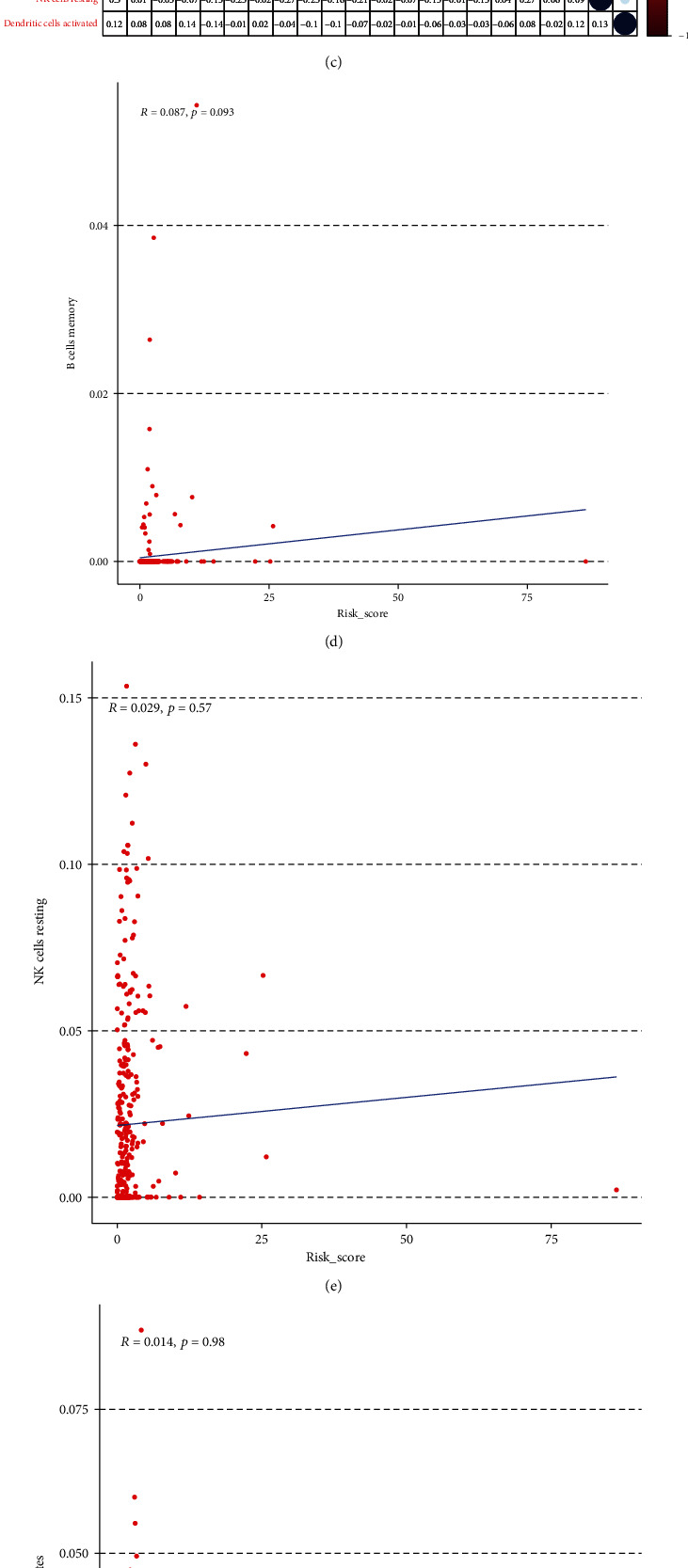
Immune infiltration of the high- and low-risk score groups and the correlation between risk score and immune cell infiltration in patients with colon cancer. (a) Bar plot showing the relative proportion of 22 kinds of infiltrating immune cells in patients from the high-risk and low-risk groups. (b) Violin plot showing the ratio differences of 22 kinds of immune cells in the high- and low- risk score samples. (c) Heatmap showing the correlation of proportions of 22 kinds of immune cells in the high- and low- risk score groups indicating the correlation between two kinds of immune cells. (d–h) Scatter plots showing the significant correlation of 5 kinds of immune cells with risk score.

The CIBERSORT algorithm was applied to further study the differences in infiltration of 22 types of immune cells in colon cancer samples. The relative content distribution of the 22 immune cell types in the colon cancer cohort and the correlation between the 22 immune cell types are shown in Figures 8(a) and 8(c).

The immune cells with significantly higher infiltration in the high-risk samples were memory B cells, resting NK cells, and monocytes (Figure 8(b)). The infiltration of macrophage M2 cells and resting dendritic cells was significantly higher in the low-risk samples than in the high-risk samples (Figure 8(b)). Correlation analyses showed that infiltration of resting dendritic cells was negatively correlation with the gene signature (Figure 8(h)), and there were no correlations between the gene signature and infiltration of memory B cells, resting NK cells, M2 macrophages cells, or monocytes (Figures 8(d)–8(g)). These data indicate that infiltration of dendritic cells is significantly correlated with the risk score and also suggest that infiltrating dendritic cells might be used as a target of biotherapy in colon cancer patients.

### 3.7. Response of High- and Low-Risk Patients to Chemotherapy, Targeted Therapy, and Immunotherapy

**Figure 9:**
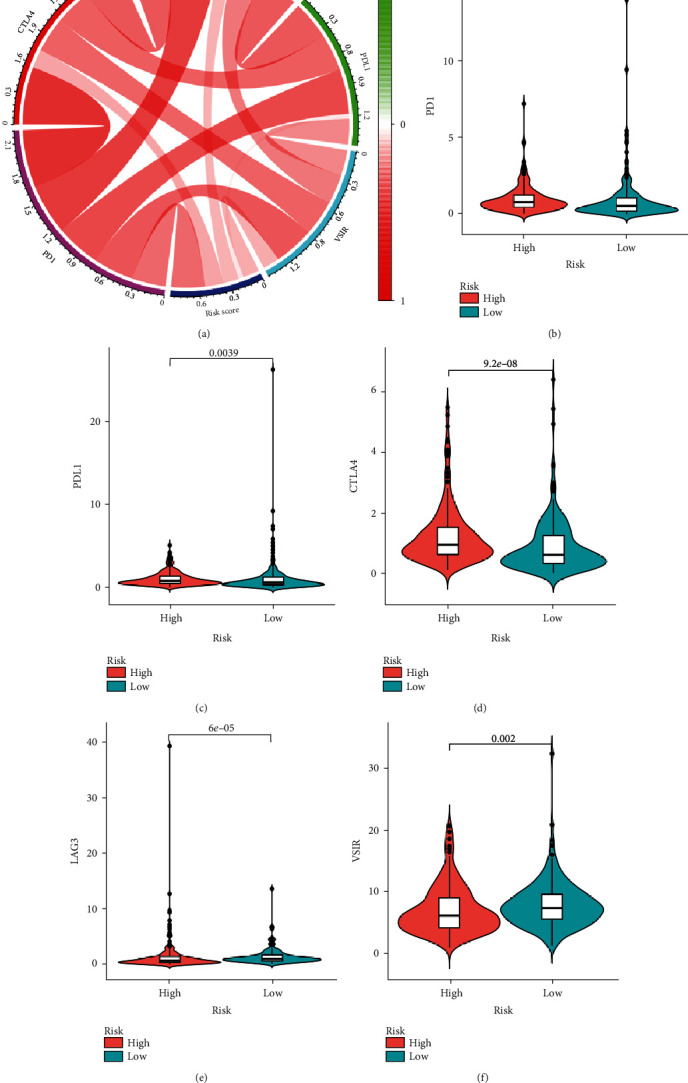
Correlation of risk scores with expression of different immune checkpoint molecules. (a) Circular plot showing correlation coefficients of risk score with expression levels of PD-1, PDL-1, CTLA-4, LAG-3, and VSIR. Cello plots showing differential expression of (b) PD-1, (c) PDL-1, (d) CTLA-4, (e) LAG-3, and (f) VSIR between the high- and low-risk score groups.

The pRRophetic algorithm was used to predict the IC50 of two common chemotherapeutic agents (cisplatin and paclitaxel) (supplementary Figure 2A-2B) and two common targeted therapeutic agents (sorafenib and sunitinib) (supplementary Figure 2C-2D) in high- and low-risk patients. There were no differences in sensitivity to cisplatin, paclitaxel, sorafenib, or sunitinib based on risk score (supplementary Figure 2A-2D). We also investigated the potential susceptibility to immune checkpoint inhibitors (targeting the immune checkpoint proteins PD-1, PDL-1, CTLA-4, LAG-3, and VSIR) in both groups. The circular plot (Figure 9(a)) showed the relationship of the risk scores and the expression of five immune checkpoint inhibitor targets.

Samples from the high-risk group had higher expression of PD-1, PDL-1, and CTLA-4 (Figures 9(b)–9(d)). The expression of LAG-3 and VSIR was significantly higher in the low-risk group (Figures 9(e) and 9(f)). These data suggest that high-risk patients may respond better to immune checkpoint inhibitors targeting PD-1, PDL-1, or CTLA-4, and low-risk patients may respond better to immune checkpoint inhibitors targeting LAG-3 or VSIR.

## 4. Discussion

The TME plays a crucial role in the pathogeneses and progression of colon cancer. Colon cancer cells regulate polarization of tumor-associated immune cells, and tumor-associated macrophages affect angiogenesis and metastasis of colon cancer. Lan et al. [17] demonstrated that M2 macrophages shuttled specific miRNAs via exosomes to colorectal cancer cells; these miRNAs targeted and downregulated BRG1 expression to promote progression of colorectal cancer. Cheng et al. [18] uncovered a new biological role for PKN2 in colon cancer as an inhibitor of M2 macrophage polarization by regulating the DUSP6-Erk1/2 pathways. Iwanaga et al. [19] demonstrated that mast cell-derived PGD2 inhibits colitis and prolongs tumor formation in colon cancer by attenuating TNF*α* signaling. Haidari et al. [20] reported that neutrophil extracellular traps play an important role in colon cancer peritoneal metastasis, migration, and invasion. Westendorf et al. [21] indicated that oxygen availability reduces CD4+ effector T cell function and suppresses the activity of Tregs to influence surveillance of inflammation-related colon cancer. Yuan and Tian [22] demonstrated that overexpression of LIN28B increases B-cell lymphoma 2 expression to promote colon cancer development. We identified significant differences in infiltration of memory B cells, resting NK cells, monocytes, M2 macrophages, and dendritic resting cells between patients stratified as low and high risk for poor outcome. These data indicate that tumor-associated immune cells may play an important role in the pathogeneses and progression of colon cancer. Therefore, it is of great interest to identify an immune-related or stroma-related signature to predict the prognosis of colon cancer patients.

In this study, we focused on the TME and established a nine-gene signature of TME-related prognostic genes. Univariate Cox analysis demonstrated that the signature was related to colon cancer stage and T, N, and M classification of colon cancer patients. Stepwise multivariate Cox analysis indicated that T classification and risk score were independent prognostic factors for colon cancer survival. The signature showed a significant ability to predict survival through risk analysis, survival analysis, and 1-year, 3-year, and 5-year multivariate ROCs. The nomogram containing age, gender, stage, and risk score showed a good practicability. Therefore, this novel prognostic signature provides an effective, practical, and quantitative method for clinicians to predict survival for patients with colon cancer.

The nine genes that composed the signature are related to the pathogeneses and progression of cancer. Miao et al. [23] demonstrated that miR-140-5p negatively regulates CEMIP and CADM3 to suppress retinoblastoma cell proliferation, migration, and invasion. Ghasemi et al. [24] found that LEP induces ovarian cancer progression. Lee et al. [25] indicated that CD1B facilitates prostate cancer progression and that low CD1B expression correlates with poorer survival. Maolake et al. [26] reported that tumor-associated macrophages activate the CCL22-CCR4 axis to promote prostate cancer migration. ABI3BP is a protective factor in gallbladder cancer, esophageal carcinoma, and lung carcinoma [27–29]. Ishimoto et al. [30] found that RHBDF2 regulates TGFB1 to induce invasion of gastric cancer by activating TACE and motility of cancer-associated fibroblasts to promote cleavage of TGFBR1. Based on the cancer relatedness of these genes, these nine genes may be potential therapeutic targets.

Dendritic cells play an important role in the pathogeneses and progression of many kinds of cancers. Zhou et al. [31] demonstrated that the maturation and function of bone marrow-derived dendritic cells are significantly inhibited by upregulation of FOXM1 through the Wnt5a signaling pathway in pancreatic cancer and colon cancer. Cheng et al. [32] demonstrated that overexpression of S100A9 protein inhibits the differentiation of dendritic cells and macrophages in cancer. Therefore, dendritic cell vaccines have been developed to treat some kinds of cancers, including colorectal cancer [33], pancreatic cancer [34], and breast cancer [35]. In our study, infiltration of dendritic cells was significantly different between the two risk groups. There were no differences in sensitivity to cisplatin, paclitaxel, sorafenib, or sunitinib between the two risk groups, but expression of the immune checkpoint molecules PD-1, PDL-1, CTLA-4, LAG-3, and VSIR was significantly different between the high- and low-risk groups. It is possible that, for colon cancer patients whose tumors are resistant to chemotherapy and immunotherapy, dendritic cell vaccines may be an effective therapeutic strategy.

The present study differs from previous studies regarding gene signatures in the colon cancer TME [36–38]. Chen and Zhao [36] constructed a TME risk score signature. However, protein expression levels of selected DEGs and the correlation between selected DEGs and immune cell infiltrations were not validated. Zhang et al. [37] identified an immune-related gene signature, but the correlation of risk score with chemotherapeutic and targeted therapeutic agents was not investigated. Ge et al. [38] indicated that genetic changes of a five-gene signature may cause loss of intestinal barrier function and translocation of gut bacteria and affect the prognosis of stage III colon cancer, but these changes were not related to immune cell infiltrations.

Here, we devised an effective, practical, and quantitative approach for clinicians to predict survival and to provide individualized treatment for patients with colon cancer. However, we recognize that there are some limitations of our current study. First, the main datasets of our study were obtained from TCGA database; other datasets should be obtained and analyzed to reduce selection bias. Second, we did not elucidate the mechanisms and function underlying the TME-related signature, which merits a further study. In addition, some crucial clinicopathological parameters, such as CEA, MSI, dMMR, and KRAS, NRAS, BRAF mutation status, were not represented in the nomogram, and it is necessary to further validate the function of this TME-related signature in additional clinical research.

## 5. Conclusions

In summary, the novel TME-related signature consisting of CADM3, LEP, CD1B, PDE1B, CCL22, ABI3BP, IGLON5, SELE, and TGFB1 is an effective, practical, and quantitative approach for clinicians to predict survival and to provide individualized treatment for colon cancer patients and is expected to be further utilized in future clinical practice.

## Figures and Tables

**Table 1:** Patient characteristics of TCGA colon cancer cohort.

Characteristic	No. of patients
Age (year)	
Median	69
<65	145
≥65	240

	
Gender	
Male	205
Female	180

Clinical stage	
I	66
II	151
III	103
IV	65

T stage	
T1	9
T2	68
T3	263
T4	44
T_X_	1

N stage	
N0	231
N1	88
N2	66

Distant metastasis	
M0	286
M1	54
Mx	45

**Table 2:** The coefficients of included genes obtained from univariate Cox regression analysis.

Gene	Coefficient	HR	HR 95%L	HR 95%H	*p* value
HOMER2	0.01459	1.48684	1.00626	2.19693	0.04644
CADM3	0.03688	1.44503	1.16200	1.79700	0.00093
PLIN4	0.04032	1.04015	1.01264	1.06841	0.00399
LEP	0.02232	1.13932	1.02569	1.26553	0.01496
HRASLS5	0.03911	1.59489	1.27205	1.99967	5.22e^−5^
CD1B	0.01789	0.04203	0.00609	0.28999	0.00129
PDE1B	0.04838	1.47214	1.11829	1.93796	0.00583
CCL22	0.00657	0.81020	0.66409	0.98847	0.03805
NGFR	0.00760	1.16778	1.06615	1.27910	0.00084
ARL4C	0.01655	1.02605	1.00802	1.04440	0.00445
ABI3BP	0.03423	1.40937	1.10454	1.79832	0.00578
FABP4	0.00798	1.01125	1.00473	1.01780	0.00068
IGLON5	0.03996	1.28561	1.13649	1.45428	6.491e^−5^
SELE	0.03134	1.27338	1.07072	1.51440	0.00628
SUSD5	0.03320	2.19843	1.49476	3.23336	6.276e^−5^
TGFB1	0.04806	1.01723	1.00007	1.03468	0.04893
NUDT10	0.03337	2.76454	1.10313	6.92817	0.03005

**Table 3:** The coefficients of included genes obtained from multivariate Cox regression analysis.

Gene	Coefficient	HR	HR 95%L	HR 95%H	*p* value
CADM3	-1.07653	0.34077	0.12161	0.95492	0.04058
LEP	0.321644	1.37939	0.99882	1.90496	0.03083
CD1B	-3.62982	0.02652	0.00247	0.28395	0.00269
PDE1B	0.958764	2.60847	1.26418	5.38220	0.00947
CCL22	-0.37199	0.68936	0.50631	0.93858	0.01815
ABI3BP	0.540378	1.71665	1.05930	2.78192	0.02824
IGLON5	0.628527	1.87484	1.17199	2.99920	0.00874
SELE	0.370045	1.44778	1.14797	1.82589	0.00177
TGFB1	0.015920	1.01604	0.99836	1.03404	0.04548

## Data Availability

The data used to support the findings of this study are included within the article.

## Supplementary Materials

Supplementary 1 Supplementary Figure 1 Association of immune and stromal and scores with clinical characteristics of colon cancer. (A-D) Distribution of immune score in age, gender, and T and N classification. (E-J) Distribution of stromal score in age, gender, T classification, N classification, M classification, and stage.

Supplementary 2 Supplementary Figure 2 Correlation of risk scores with chemotherapeutics such as cisplatin (A), paclitaxel (B), sorafenib (C), and sunitinib (D).
