# Supplementary figures and images for: Identification of a Tumor Microenvironment-Related Gene Signature Indicative of Disease Prognosis and Treatment Response in Colon Cancer
Source: Oxid Med Cell Longev. 2021 Aug 14;2021:6290261. doi: 10.1155/2021/6290261 (PMC8420973; doi:10.1155/2021/6290261)

**A**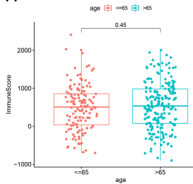**B**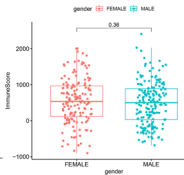**C**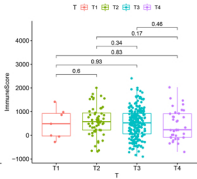**D**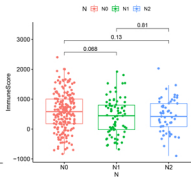**E**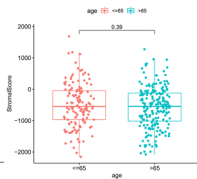**F**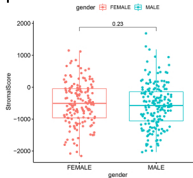**G**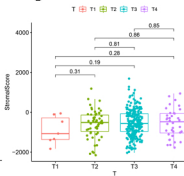**H**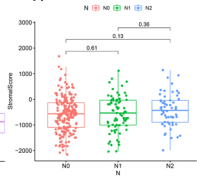**I**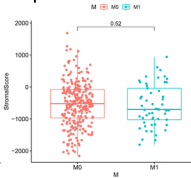**J**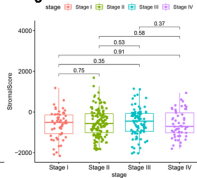

Supplement: Supplementary 1 — Supplementary Figure 1 Association of immune and stromal and scores with clinical characteristics of colon cancer. (A-D) Distribution of immune score in age, gender, and T and N classification. (E-J) Distribution of stromal score in age, gender, T classification, N classification, M classification, and stage. [file 6290261.f1.pdf]

**A**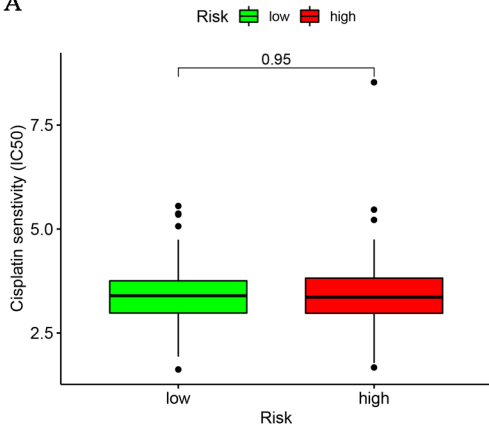**B**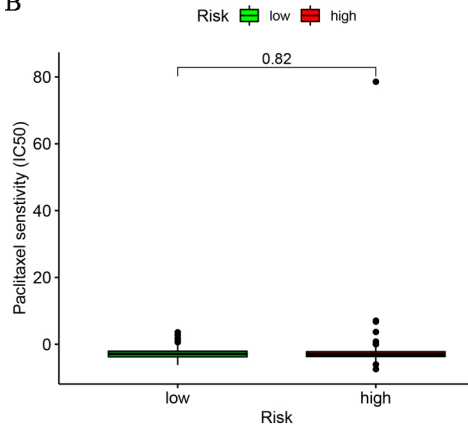**C**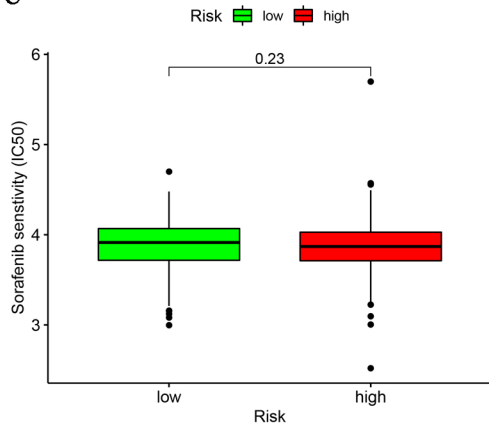**D**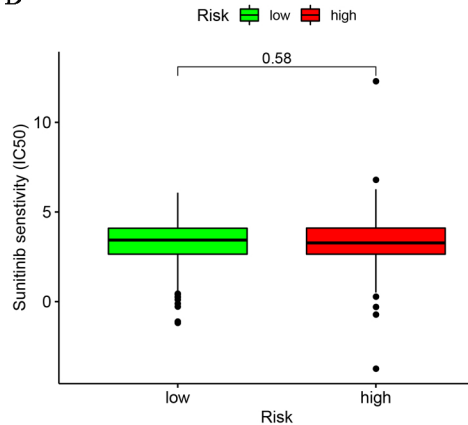

Supplement: Supplementary 2 — Supplementary Figure 2 Correlation of risk scores with chemotherapeutics such as cisplatin (A), paclitaxel (B), sorafenib (C), and sunitinib (D). [file 6290261.f2.pdf]
